# Supplementary material for: RNA-Guided AsCas12a- and SpCas9-Catalyzed Knockout and Homology Directed Repair of the Omega-1 Locus of the Human Blood Fluke, Schistosoma mansoni
Source: Int J Mol Sci. 2022 Jan 6;23(2):631. doi: 10.3390/ijms23020631 (PMC8775552; doi:10.3390/ijms23020631)
Supplement: Supplementary file 1 [file ijms-23-00631-s001.zip › ijms-1520961-supplementary_Jan 12, 2022.pdf]

International Journal of *Molecular Sciences*

MDPI

Supplementary Materials:

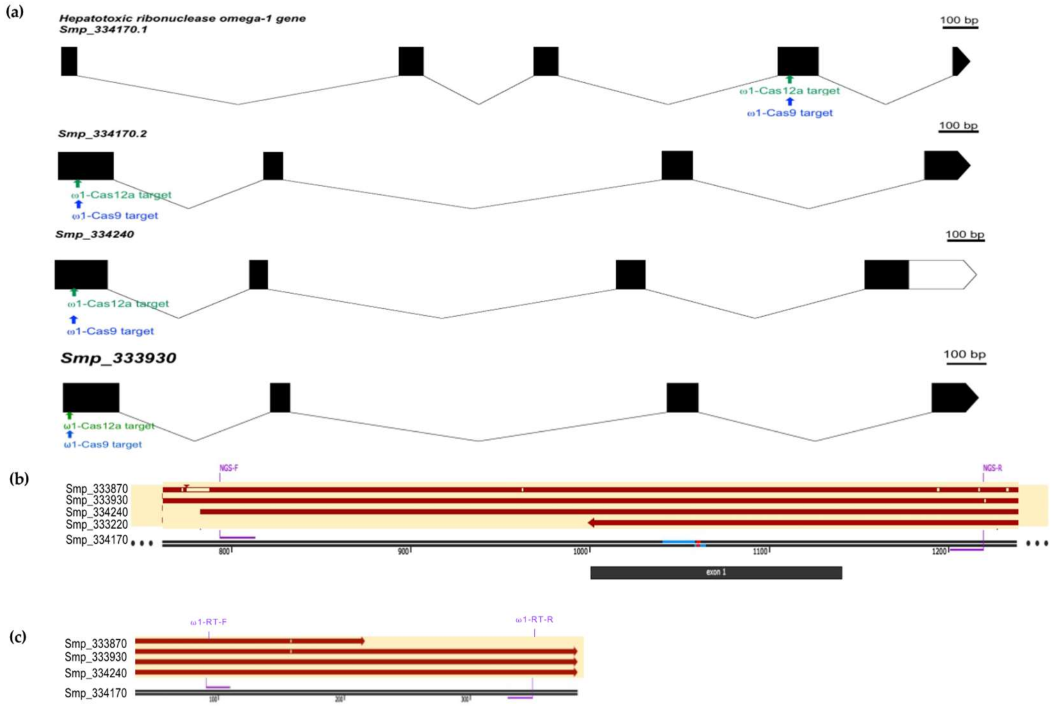

Figure S1. Gene structure encoding the hepatotoxic ribonuclease, omega 1 (*ω1*). At least five copies of the gene are located on chromosome 1, as apparent in the draft sequence of *S. mansoni*, version 7, scaffold SM_V7_1:3,885,722-4,002,365. The positions of the several *ω1* genes of the schistosome genome (WormBase Parasite, September 2021), including two loci of Smp_334170, Smp_334240, Smp_334070, Smp_3332220, Smp_333930, Smp_333870 and Smp_179960 are shown to for their physical relationship of the copies on the chromosome: **(a)** Gene structures and sizes of Smp_334170.1, Smp_334170.2 and Smp_334240 and the CRISPR/Cas programmed target sites for *Sp*Cas9 (blue arrow) and *As*Cas12a (green); **(b)** All gene copies of *omega-1* share >99% DNA sequence identity; DNA sequence alignment of the *omega-1* orthologues and target amplicon primer indicated as NGS-F and NGS-R. Four copies of the gene, Smp_334240, Smp_334170 and Smp_333930, are reported in Uniprot; **(c)** Alignment of mRNA sequences and primers used for gene transcript analysis (purple).

mixed gide RNA only	amount of reads (%)		
	reference	Smp_333930	Smp_333870
unmodified	202009 (80.09%)	25262 (10.77%)	2197 (0.94%)
modified	2266 (0.97%)	2108 (0.89%)	265 (0.11%)
ambibuous	538 (0.23%)		
**Cas9 only**	**amount of reads (%)**		
	**reference**	**Smp_333930**	**Smp_333870**
unmodified	197631 (83.35%)	26097 (10.71%)	5989 (2.46%)
modified	1116 (0.47%)	3235 (1.33%)	679 (0.28%)
ambibuous	4262 (1.85%)		
**Cas12a only**	**amount of reads (%)**		
	**reference**	**Smp_333930**	**Smp_333870**
unmodified	182751 (79.53%)	26097 (10.71%)	5989 (2.46%)
modified	4775 (2.08%)	3235 (1.33%)	679 (0.28%)
ambibuous	4262 (1.85%)		
**mixed donor only**	**amount of reads (%)**		
	**reference**	**Smp_333930**	**Smp_333870**
unmodified	180841 (83.73%)	21237 (9.83%)	5321 (2.46%)
modified	1000 (0.46%)	3735 (1.73%)	773 (0.36%)
ambibuous	3083 (1.43%)		

Table S1. The CRISPREsso2 analysis findings from four negative controls: mixed sgRNAs only, *Sp*Cas9 only, *As*Cas12a only, and mixed donors only. There were over 180,000 reads from each library. There was < 1% of background modified reads from all negative control samples.

Primer name	5' to 3' sequence	Expected amplicon size (bp)	note
control-F	tattgtcaacggcgtacagg	790	pair with control-R
control-R	ctggaatcatgtggtgagatcc		
NGS-F	tattgtcaacggcgtacagg	426	pair with NGS-R
NGS-R	caatcggcactgagacgca		
5'-int-F	tatgataaggcgttttgtgctg	235	pair with 24nt-6stp-R
24nt-6stp-R	gctactcagttacctagtcactta		
24nt-6st-F	taagtgactggtaactgagtagc	182	pair with 3'-int-R
3'-int-R	caatcggcactgagacgca		
ω1-RT-F	ggttactttaaatttggta	259	pair with ω1-RT-R
ω1-RT-R	agtttccaaggaacgggcag		
Sm GAPDH-F	atgggacatttccaggcgag	285	pair with *Sm* GAPDH-F
Sm GAPDH-R	ccaacaacgaacatgggtgc		

Table S2. Nucleotide sequences of oligonucleotide primers used in the investigation.
